# Global microbial community biodiversity increases with antimicrobial toxin abundance of rare taxa

**DOI:** 10.1093/ismejo/wraf012

**Published:** 2025-01-24

**Authors:** Ya Liu, Yu Geng, Yiru Jiang, Peng Li, Yue-zhong Li, Zheng Zhang

**Affiliations:** State Key Laboratory of Microbial Technology, Institute of Microbial Technology, Shandong University, Qingdao 266237, China; Qilu Hospital (Qingdao), Cheeloo College of Medicine, Shandong University, Qingdao 266035, China; Suzhou Research Institute, Shandong University, Suzhou 215123, China; State Key Laboratory of Microbial Technology, Institute of Microbial Technology, Shandong University, Qingdao 266237, China; State Key Laboratory of Microbial Technology, Institute of Microbial Technology, Shandong University, Qingdao 266237, China; State Key Laboratory of Microbial Technology, Institute of Microbial Technology, Shandong University, Qingdao 266237, China; State Key Laboratory of Microbial Technology, Institute of Microbial Technology, Shandong University, Qingdao 266237, China; State Key Laboratory of Microbial Technology, Institute of Microbial Technology, Shandong University, Qingdao 266237, China

**Keywords:** microbial community, antimicrobial toxin, biodiversity, rare taxa, Earth Microbiome Project, antagonism

## Abstract

One of the central questions in microbial ecology is how to explain the high biodiversity of communities. A large number of rare taxa in the community have not been excluded by abundant taxa with competitive advantages, a contradiction known as the biodiversity paradox. Recently, increasing evidence has revealed the central importance of antimicrobial toxins as crucial weapons of antagonism in microbial survival. The powerful effects of antimicrobial toxins result in simple combinations of microorganisms failing to coexist under laboratory conditions, but it is unclear whether they also have a negative impact on the biodiversity of natural communities. Here, we revealed that microbial communities worldwide universally possess functional potential for antimicrobial toxin production. Counterintuitively, the biodiversity of global microbial communities increases, rather than decreases, as the abundance of antimicrobial toxins in rare taxa rises. Rare taxa may encode more antimicrobial toxins than abundant taxa, which is associated with the maintenance of the high biodiversity of microbial communities amid complex interactions. Our findings suggest that the antagonistic interaction caused by antimicrobial toxins may play a positive role in microbial community biodiversity at the global scale.

Explaining how the tremendous biodiversity in natural communities persists despite differences between species in competitive ability is essential in microbial ecology [1, 2]. Microbial communities typically exhibit a skewed abundance distribution, with a small number of competitively abundant taxa coexisting alongside a high number of low-abundance rare taxa [3, 4]. Antagonistic interactions among microorganisms in communities are prevalent, and a mathematical model predicts that communities dominated by antagonism rather than cooperation are more stable [5, 6]. Increasing evidence emphasizes the central role of antagonism in microbial life, but our understanding of how it affects the coexistence of abundant taxa and rare taxa, as well as how it shapes community biodiversity, remains limited [7].

Antimicrobial toxins serve as crucial weapons in intermicrobial antagonism, helping microbes displace competitors to occupy spatial and nutritional niches in communities [8]. They can be secreted through cell contact-dependent (e.g. type VI secretion systems) and contact-independent (e.g. bacteriocins) manners, requiring only minute amounts to disrupt key molecular structures in target cells [9]. The selective pressure of antagonism drives a continuous arms race between antimicrobial toxin weapons and defense systems in communities [10, 11]. All major bacterial phyla have been shown to encode antimicrobial toxin genes (ATGs), and many taxa deploy a diverse arsenal [12]. The potent effects of antimicrobial toxins may result in simple combinations of different microorganisms failing to coexist under laboratory conditions, with high biodiversity emerging as a property of community assembly [1, 13]. However, the relationship between antimicrobial toxins and biodiversity in natural microbial community remains understudied.

The genome-sequenced proportion of global microbial communities has reached a high level [14, 15]. Here, we identified ATG compositions for amplicon sequence variants (ASVs) included in the 10 000 samples released by the Earth Microbiome Project (EMP) dataset [3] and calculated the ATG abundance for each sample (Supplementary Methods and Fig. 1A). For this purpose, we first mapped the 16S ribosomal RNA (rRNA) gene sequences to sequenced genomes with a 100% identity threshold and then identified ATGs in the resulting 6019 genomes (Fig. S1 and Table S1). A total of 149 antimicrobial toxin domain families, 73 immunity protein families, 42 secretion-related marker domain families (such as trafficking domains, repeat domains, protoxins, and conserved motifs), and 4 adaptor families were collected from the literature, and genes encoding these domains were searched in the genomes. We screened candidate ATGs that encoded N-terminal secretion-related markers or were adjacent to genes encoding immunity proteins, and further ensured that each identified ATG contained secretion features. Our results show that for the representative communities from typical habitats worldwide, almost all (99.8%) contained identifiable ATGs (Table S2). The median ATG abundance in the communities was 0.629 (0.322–0.954) genes/cell, and the median biodiversity was 236 (106–505) observed ASVs (Figs 1B and S2). Counterintuitively, although antimicrobial toxins mediate intermicrobial killing, there was a weak but significant positive correlation between biodiversity and ATG abundance in global microbial communities (Spearman’s *ρ* = 0.134, *P* < 0.0001).

**Figure 1:**
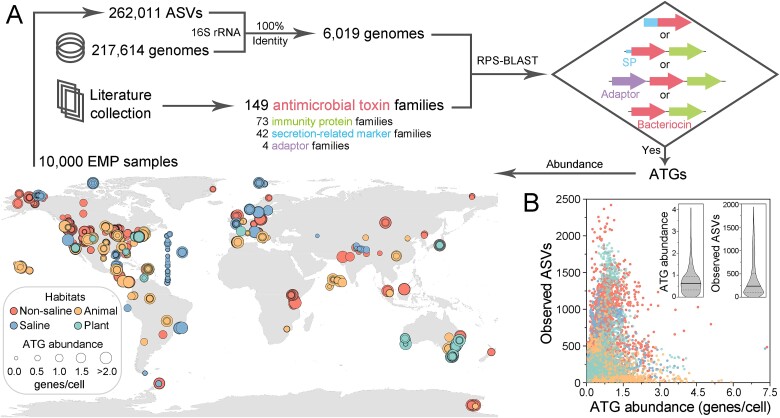
Antimicrobial toxin genes (ATGs) were ubiquitous in microbial communities worldwide. (A) Estimation of ATG abundance in microbial communities (see supplementary methods). Analysis was conducted using a subset of 10 000 samples released by the EMP. The 16S rRNA gene sequences were mapped to sequenced genomes with a 100% identity threshold, and then features such as gene neighborhoods, domain architectures, and others were used to identify ATGs in the resulting genomes. The ATG abundance (genes/cell) was estimated based on the number of ATGs and 16S rRNA genes encoded by the genomes within a community. Samples are displayed on a world map according to their latitudinal and longitudinal coordinates, with dot sizes representing the ATG abundance. (B) ATG abundance and biodiversity of the studied microbial communities. The analysis was based on 7368 microbial communities representing typical habitats worldwide. Each dot represents a community, with colors indicating different habitat types. The middle line inside the violin plots shows the median, and the dashed line represents the 25^th^–75^th^ percentiles.

Microbial communities typically comprise a low number of competitively abundant taxa and a high number of rare taxa (Fig. S3). We found that the ATG abundance in rare taxa was significantly greater than that in abundant taxa in global microbial communities (Wilcoxon signed-rank test, *P* < 0.0001; Fig. 2A and Fig. S4). This phenomenon remained consistent even when the abundance thresholds for defining rare and abundant taxa were adjusted (Fig. S5 and Table S3). Furthermore, ATG abundance was influenced by two factors: the proportion of ATG-encoding cells in all cells and the average number of ATGs per ATG-encoding cell. Rare taxa exhibited significantly greater values for both factors than abundant taxa (Fig. 2B). Moreover, as the ATG abundance of rare taxa increased, both the ASV proportion and the abundance proportion of rare taxa in the community also significantly increased (Fig. 2C and Fig. S6).

**Figure 2:**
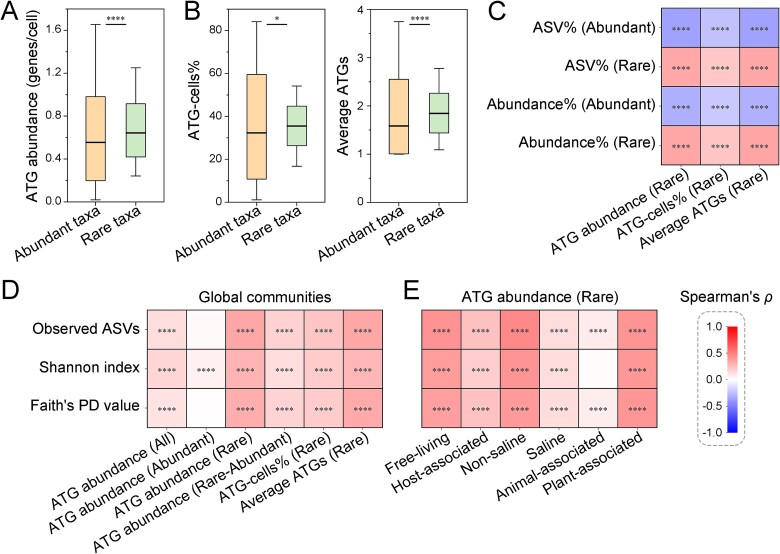
Biodiversity of microbial communities increased with the ATG abundance of rare taxa at the global scale. (A) ATG abundance of abundant and rare taxa in microbial communities. Within a community, ASVs with relative abundances of ≥1% of the total sequences were regarded as “abundant”, and those with relative abundances of <0.1% were designated “rare”. (B) Proportion of ATG-encoding cells in all cells (ATG-cells%) and the average number of ATGs per ATG-encoding cell (average ATGs) in rare and abundant taxa. (C) Correlations between ATG abundance of rare taxa and the proportion of rare and abundant taxa in the communities. ASV% (abundant) represents the proportion of ASVs from abundant taxa within the community relative to the total number of ASVs. Abundant% (abundant) represents the proportion of the sum of the relative abundances from abundant taxa to the total sequence within the community. The same applies to the proportion of rare taxa. (D) Correlations between microbial community biodiversity and ATG abundance. Biodiversity indices include observed ASVs, Shannon index, and Faith’s PD value. The ATG abundance was calculated separately for all taxa, abundant taxa, and rare taxa within the community. Additionally, the ATG abundance difference between rare and abundant taxa was analyzed, along with two factors that influence the ATG abundance of rare taxa. (E) Correlations between the ATG abundance in rare taxa and community biodiversity across different habitats. Global microbial communities were classified as free-living and host-associated, with further subdivision into four types of communities: Non-saline, saline, animal-associated, and plant-associated. For the box plots, the middle line indicates the median, the box represents the 25^th^–75^th^ percentiles, and the error bar indicates the 10^th^–90^th^ percentiles of observations. Comparisons between bins were analysed via the Wilcoxon signed-rank test. ^*^P < 0.05; ^*^^*^^*^^*^P < 0.0001.

Regardless of the index used to characterize community biodiversity, biodiversity was consistently significantly positively correlated with the ATG abundance of rare taxa but showed little to no correlation with the ATG abundance of abundant taxa (Fig. 2D and Fig. S7). The greater the ATG abundance difference between rare taxa and abundant taxa in communities, the higher the biodiversity. Although biodiversity varied significantly across different habitats (Fig. S8), a positive correlation between the ATG abundance of rare taxa and community biodiversity was observed in both the free-living communities (including non-saline and saline habitats) and the host-associated communities (including animal-associated and plant-associated habitats) (Fig. 2E and Fig. S9). These findings lead us to conclude that microbial community biodiversity increases with the abundance of antimicrobial toxins in rare taxa on a global scale.

The antagonistic potential of microorganisms largely relies on the collection of ATGs contained in their genomes [5, 7]. Our results indicate that antimicrobial toxins are universally encoded across global microbial communities. However, rare taxa within these communities may possess stronger antimicrobial weapons than abundant taxa, which is associated with the stable coexistence of species in highly diverse communities. This phenomenon appears to be distinct from the mechanisms underpinning persistence through positive interactions between rare species in animal or plant communities [16]. Several mechanisms may explain how antimicrobial toxins contribute to maintaining high biodiversity: increasing community biodiversity by promoting spatial segregation [17–19] or strengthening interaction networks to help stabilize multispecies coexistence [20, 21]. However, the mere presence of ATGs does not necessarily imply their functional expression in the environments. Future comprehensive analysis of community interactions may elucidate the detailed mechanisms by which rare taxa harboring more ATGs contribute to the enhancement of biodiversity. In conclusion, our study supports the view that high biodiversity is an emergent property of community assembly, and highlights the central importance of antimicrobial toxin-mediated antagonism in microbial communities. Understanding the close relationship between ATG abundance and community biodiversity has significant implications for those aiming to manipulate and engineer microbial communities for human benefit.

## Supplementary Material

Supplementary_Information_wraf012

Supplementary_Table_1_wraf012

Supplementary_Table_2_wraf012

Supplementary_Table_3_wraf012

## Data Availability

Data that support the findings of this study are available in the supplementary information. Source data are provided with this paper.
