# Supplementary material for: Global microbial community biodiversity increases with antimicrobial toxin abundance of rare taxa
Source: ISME J. 2025 Jan 24;19(1):wraf012. doi: 10.1093/ismejo/wraf012 (PMC11822679; doi:10.1093/ismejo/wraf012)
Supplement: Supplementary_Information_wraf012 [file supplementary_information_wraf012.pdf]

Supplementary Information

Global microbial community biodiversity increases with antimicrobial toxin abundance of rare taxa

Ya Liu[1, 2, 3], Yu Geng[1], Yiru Jiang[1], Peng Li[1], Yue-zhong Li[1, *], Zheng Zhang[1, *]

[1] *State Key Laboratory of Microbial Technology, Institute of Microbial Technology, Shandong University, Qingdao 266237, China*

[2] *Qilu Hospital (Qingdao), Cheeloo College of Medicine, Shandong University, Qingdao 266035, China*

[3] *Suzhou Research Institute, Shandong University, Suzhou 215123, China*

*Address correspondence to Yue-zhong Li (E-mail: lilab@sdu.edu.cn, ORCID: 0000-0001-8336-6638) or Zheng Zhang (E-mail: zhangzheng@sdu.edu.cn, ORCID: 0000-0001-9971-6006)

The supplementary information includes the following:

Supplementary Methods

Figures S1-S9

Legends for Tables S1-S3

Supplementary Methods

Data collection of microbial communities

The Earth Microbiome Project (EMP) is a massively collaborative effort aimed at understanding patterns in microbial ecology across communities and habitats on Earth [1]. The analysis consisted of a 10,000-sample subset published by the EMP, which was carefully chosen to ensure the representation of different habitat types and relevance to various research studies. A total of 262,011 amplicon sequence variants (ASVs), their abundance, and nucleic acid sequence information were collected, which were obtained and shared by the EMP from 10,000 samples using Deblur software [2]. All samples were categorized into free-living or host-associated communities, which were further divided into four categories: saline (free-living), non-saline (free-living), animal (host-associated) and plant (host-associated).

The biodiversity of microbial communities includes observed ASVs, the Shannon index, and Faith's PD (phylogenetic diversity) value [3,4]. Microbial community biodiversity differed significantly across habitats (**Fig. S8**). Abundant taxa were defined as ASVs with a relative abundance ≥ 1% of the total sequences within a sample, whereas rare taxa were defined as ASVs with a relative abundance < 0.1% [5]. Abundant ASVs accounted for only 7.1% (3.0%-14.3%) of the total number of ASVs in the community, but accounted for 72.8% (51.5%-87.0%) of the total 16S rRNA gene abundance in the community. In contrast, rare ASVs comprise 62.5% (48.6%-76.3%) of the total number of ASVs, but contribute only 5.1% (1.9%-12.7%) of the total 16S rRNA gene

abundance in the community (**Fig. S3**).

Acquisition of genetic information in communities

The NCBI reference sequence (RefSeq) database is a curated non-redundant collection of sequences representing whole or frame genomes [6]. We acquired a dataset consisting of 217,614 sourced bacterial or archaeal genomes. The ASVs from the EMP data were mapped to the sequenced genomes, with a 100% identity threshold in the 16S rRNA gene (V4 region) for better discrimination of closely related organisms. A total of 6,019 sequenced prokaryotic genomes were included in the EMP samples, 98% of which were from bacteria and 2% from archaea. Subsequent analysis was conducted only on samples (a total of 7,368 samples) meeting the following criteria: the percentage of ASVs and the percentage of sequence abundance mapped to the genome were both greater than 5%, and there were at least three ASVs from abundant taxa and at least three ASVs from rare taxa. In the studied samples, the median proportions of cells and taxa with known genomic information were 52.5% (30.1%-91.6%) and 27.9% (14.5%-62.9%), respectively (**Fig. S1**). This indicates that in more than half of the microbial communities, genomic information for at least 52% of cells and 27% of ASVs has been reported. When analyzed under a 97% sequence identity threshold, the median proportions of cells and taxa with known genomic information increased to 73.2% (55.7%-96.3%) and 49.2% (34.7%-79.4%), respectively, across the 7,368 samples (**Fig. S2**). Compared with those reported in previous studies, the proportion of sequenced genomes in global microbial communities has further increased [7].

ATG identification in genomes

Sequence information on reported antimicrobial toxin proteins, immunity proteins, secretion-related markers and adaptors was collected from the literature [8]. Secretion-related markers contain trafficking domains, repeat domains, pre-toxins, and conserved motifs [9-11]. Trafficking domains include, for example, VgrG [12], PAAR [13,14], LXG [15], DUF4157 [16], WXG100 [17], SpvB [11], TANFOR [18], Phage_Mu_F [11], and FhaB [19]. Repeat domains include Haemagg_act [20], RHS repeat [21], and so on. Pre-toxins include PT-HINT [22], PT-TG [22], PT-VENN [23], and others. Conserved motifs include Mix [24] and Fix [25]. The DUF4123 [26], DUF2169 [27], DUF1795 [28] and PRK06147 [29] protein families are described as adaptors. A total of 149 antimicrobial toxin families, 73 immunity protein families, 42 secretion-related marker families and 4 adaptor families were identified (**Table S1**).

All genes encoding the aforementioned antimicrobial toxin families, immunity protein families, secretion-related marker families and adaptor families were scanned among the 6,019 sequenced prokaryotic genomes contained in the EMP samples by RPS-BLAST (expected value threshold 0.01) [30]. On this basis, the domain architectures and gene neighborhoods of antimicrobial toxin families (candidate ATGs) were further analysed. If a candidate ATG satisfies one of the four conditions, it is determined that the gene encodes an ATG: i) the N-terminus of the protein product encoded by the gene has at least one secretion-related marker domain; ii) the downstream neighborhood of the gene encodes a corresponding immunity protein, with the N-terminus encoding a signal peptide; iii) the upstream neighboring gene also encodes an adaptor in addition

to the downstream neighborhood of the gene encoding the corresponding immunity protein; and iv) the gene encodes bacteriocin families and its downstream neighborhood encodes the corresponding immunity protein. Ultimately, a total of 4,774 ATGs were identified (**Table S1**). The need for genome information under these stringent criteria compelled us to use a 16S rRNA gene mapping-based approach rather than a metagenomic approach to assess the abundance of ATGs in the community. Additionally, the presence of ATGs in the communities implies the functional potential to produce antimicrobial toxins but does not necessarily indicate their actual expression in the environment.

Calculation of ATG abundance in communities

The relative abundance of ATGs in each community was calculated based on the number of ATGs and 16S rRNA genes encoded by the mapped genomes in the community (**Table S2**), representing the copy number of ATGs normalized against the cell number (genes/cell):

$$\text{ATG abundance} = \frac{\sum_{i=1}^{n} N_{i\text{ATG}} \times R_i / N_{i\text{16S}}}{\sum_{j=1}^{m} R_j / N_{j\text{16S}}}$$

Here, *m* is the total number of ASVs mapped to the genomes in the community; *n* is the total number of ASVs with genome-encoded ATGs; *i* and *j* represent specific ASVs in the community; R_i represents the relative abundance of the ASV; $N_{i\text{ATG}}$ represents the number of ATGs in the genome; and $N_{i\text{16S}}$ represents the number of 16S rRNA genes in the genome. Due to biases introduced during PCR amplification, the cell counts here do not directly correspond to the actual cell numbers in the community.

The ATG abundance in the community was influenced by two factors: (1) the proportion of ATG-encoding cells in all cells (ATG-cells%) and (2) the average number of ATGs per ATG-encoding cell (average ATGs). We performed calculations for each of these factors separately:

$$\text{ATG-cells\%} = \frac{\sum_{i=1}^{n} R_i / N_{i\mathrm{16S}}}{\sum_{j=1}^{m} R_j / N_{j\mathrm{16S}}} \times 100\%$$

$$\text{average ATGs} = \frac{\sum_{i=1}^{n} N_{i\mathrm{ATG}} \times R_i / N_{i\mathrm{16S}}}{\sum_{i=1}^{n} R_i / N_{i\mathrm{16S}}}$$

The ATG abundance, ATG-cells%, and average ATGs were calculated separately for both abundant and rare microbial taxa within the community. For comparison, we also calculated the ATG abundance based on different thresholds for abundant taxa (ASVs with a relative abundance ≥ 2%, ≥ 1%, ≥ 0.9%, ≥ 0.8%, ≥ 0.7%, or ≥ 0.6% within a sample) and rare taxa (ASVs with a relative abundance < 0.05%, < 0.1%, < 0.2%, < 0.3%, < 0.4%, or < 0.5% within a sample) (**Fig. S5 and Table S3**).

The ATG abundance was not significantly correlated with the proportion of cells (P = 0.662) or taxa (P = 0.003) with known genomic information in the communities (**Fig. S1**). We also mapped the 16S rRNA gene sequences to sequenced genomes using a 97% sequence identity threshold and calculated the ATG abundance (**Fig. S2**). The ATG abundances calculated under both conditions were highly positively correlated (R^2 = 0.82, P < 0.001), and both were positively correlated with community biodiversity (**Fig. S4**).

Statistical analysis

Univariate associations were determined using Spearman's rank correlation. For all comparisons, the Wilcoxon signed-rank test was used to compare differences between paired samples, and the Wilcoxon rank-sum test was used to compare differences between independent samples. Unless otherwise stated, *$P < 0.05$; **$P < 0.01$; ***$P < 0.001$; ****$P < 0.0001$.

References

1. Thompson LR, Sanders JG, McDonald D *et al.* A communal catalogue reveals Earth's multiscale microbial diversity. *Nature* 2017;**551**:457-463. https://doi.org/10.1038/nature24621
2. Amir A, McDonald D, Navas-Molina JA *et al.* Deblur rapidly resolves single-nucleotide community sequence patterns. *mSystems* 2017;**2**:e00191-16. https://doi.org/10.1128/mSystems.00191-16
3. Shannon CE. A mathematical theory of communication. *Bell Syst Tech J* 1948;**27**:379-423. https://doi.org/10.1002/j.1538-7305.1948.tb01338.x
4. Faith DP. Conservation evaluation and phylogenetic diversity. *Biol Conserv* 1992;**61**:1-10. https://doi.org/10.1016/0006-3207(92)91201-3
5. Pedrós-Alió C. The rare bacterial biosphere. *Annu Rev Mar Sci* 2012;**4**:449-466. https://doi.org/10.1146/annurev-marine-120710-100948
6. Li WJ, O'Neill KR, Haft DH *et al.* RefSeq: expanding the Prokaryotic Genome

Annotation Pipeline reach with protein family model curation. *Nucleic Acids Res* 2021;**49**:D1020-D1028. https://doi.org/10.1093/nar/gkaa1105

7. Zhang Z, Wang JN, Wang JL *et al.* Estimate of the sequenced proportion of the global prokaryotic genome. *Microbiome* 2020;**8**:134. https://doi.org/10.1186/s40168-020-00903-z
8. Liu Y, Liu S, Pan Z *et al.* PAT: a comprehensive database of prokaryotic antimicrobial toxins. *Nucleic Acids Res* 2023;**51**:D452-D459. https://doi.org/10.1093/nar/gkac879
9. Ruhe ZC, Low DA, Hayes CS. Polymorphic toxins and their immunity proteins: diversity, evolution, and mechanisms of delivery. *Annu Rev Microbiol* 2020;**74**:497-520. https://doi.org/10.1146/annurev-micro-020518-115638
10. Makarova KS, Wolf YI, Karamycheva S *et al.* Antimicrobial peptides, polymorphic toxins, and self-nonself recognition systems in archaea: an untapped armory for intermicrobial conflicts. *mBio* 2019;**10**:e00715-19. https://doi.org/10.1128/mBio.00715-19
11. Zhang DP, de Souza RF, Anantharaman V *et al.* Polymorphic toxin systems: Comprehensive characterization of trafficking modes, processing, mechanisms of action, immunity and ecology using comparative genomics. *Biol Direct* 2012;**7**:18. https://doi.org/10.1186/1745-6150-7-18
12. Mougous JD, Cuff ME, Raunser S *et al.* A virulence locus of *Pseudomonas aeruginosa* encodes a protein secretion apparatus. *Science* 2006;**312**:1526-1530. https://doi.org/10.1126/science.1128393

13. Shneider MM, Buth SA, Ho BT *et al.* PAAR-repeat proteins sharpen and diversify the type VI secretion system spike. *Nature* 2013;**500**:350-353. https://doi.org/10.1038/nature12453

14. Zhang Z, Liu Y, Zhang P *et al.* PAAR proteins are versatile clips that enrich the antimicrobial weapon arsenals of prokaryotes. *mSystems* 2021;**6**:e00953-21. https://doi.org/10.1128/mSystems.00953-21

15. Whitney JC, Peterson SB, Kim J *et al.* A broadly distributed toxin family mediates contact-dependent antagonism between gram-positive bacteria. *eLife* 2017;**6**:e26938. https://doi.org/10.7554/eLife.26938

16. Geller AM, Pollin I, Zlotkin D *et al.* The extracellular contractile injection system is enriched in environmental microbes and associates with numerous toxins. *Nat Commun* 2021;**12**:3743. https://doi.org/10.1038/s41467-021-23777-7

17. Pallen MJ. The ESAT-6/WXG100 superfamily–and a new Gram-positive secretion system? *Trends Microbiol* 2002;**10**:209-212. https://doi.org/10.1016/s0966-842x(02)02345-4

18. Jana B, Salomon D, Bosis E. A novel class of polymorphic toxins in Bacteroidetes. *Life Sci Alliance* 2020;**3**:e201900631. https://doi.org/10.26508/lsa.201900631

19. Locht C, Geoffroy MC, Renauld G. Common accessory genes for the *Bordetella pertussis* filamentous hemagglutinin and fimbriae share sequence similarities with the *papC* and *papD* gene families. *EMBO J* 1992;**11**:3175-3183. https://doi.org/10.1002/j.1460-2075.1992.tb05394.x

20. Kajava AV, Cheng N, Cleaver R *et al.* Beta-helix model for the filamentous

haemagglutinin adhesin of *Bordetella pertussis* and related bacterial secretory proteins. *Mol Microbiol* 2001;**42**:279-292. https://doi.org/10.1046/j.1365-2958.2001.02598.x

21. Busby JN, Panjikar S, Landsberg MJ *et al.* The BC component of ABC toxins is an RHS-repeat-containing protein encapsulation device. *Nature* 2013;**501**:547-550. https://doi.org/10.1038/nature12465

22. Zhang DP, Iyer LM, Aravind L. A novel immunity system for bacterial nucleic acid degrading toxins and its recruitment in various eukaryotic and DNA viral systems. *Nucleic Acids Res* 2011;**39**:4532-4552. https://doi.org/10.1093/nar/gkr036

23. Aoki SK, Diner EJ, de Roodenbeke CT *et al.* A widespread family of polymorphic contact-dependent toxin delivery systems in bacteria. *Nature* 2010;**468**:439-442. https://doi.org/10.1038/nature09490

24. Salomon D, Kinch LN, Trudgian DC *et al.* Marker for type VI secretion system effectors. *Proc Natl Acad Sci USA* 2014;**111**:9271-9276. https://doi.org/10.1073/pnas.1406110111

25. Jana B, Fridman CM, Bosis E *et al.* A modular effector with a DNase domain and a marker for T6SS substrates. *Nat Commun* 2019;**10**:3595. https://doi.org/10.1038/s41467-019-11546-6

26. Liang XY, Moore R, Wilton M *et al.* Identification of divergent type VI secretion effectors using a conserved chaperone domain. *Proc Natl Acad Sci USA* 2015;**112**:9106-9111. https://doi.org/10.1073/pnas.1505317112

27. Unterweger D, Kostiuk B, Pukatzki S. Adaptor proteins of type VI secretion system effectors. *Trends Microbiol* 2017;**25**:8-10. https://doi.org/10.1016/j.tim.2016.10.003

28. Whitney JC, Quentin D, Sawai S *et al.* An interbacterial NAD(P)$^+$ glycohydrolase toxin requires elongation factor Tu for delivery to target cells. *Cell* 2015;**163**:607-619. https://doi.org/10.1016/j.cell.2015.09.027

29. Liu Y, Zhang Z, Wang F *et al.* Identification of type VI secretion system toxic effectors using adaptors as markers. *Comput Struct Biotechnol J* 2020;**18**:3723-3733. https://doi.org/10.1016/j.csbj.2020.11.003

30. Wang J, Chitsaz F, Derbyshire MK *et al.* The conserved domain database in 2023. *Nucleic Acids Res* 2023;**51**:D384-D388. https://doi.org/10.1093/nar/gkac1096

Supplementary Figures

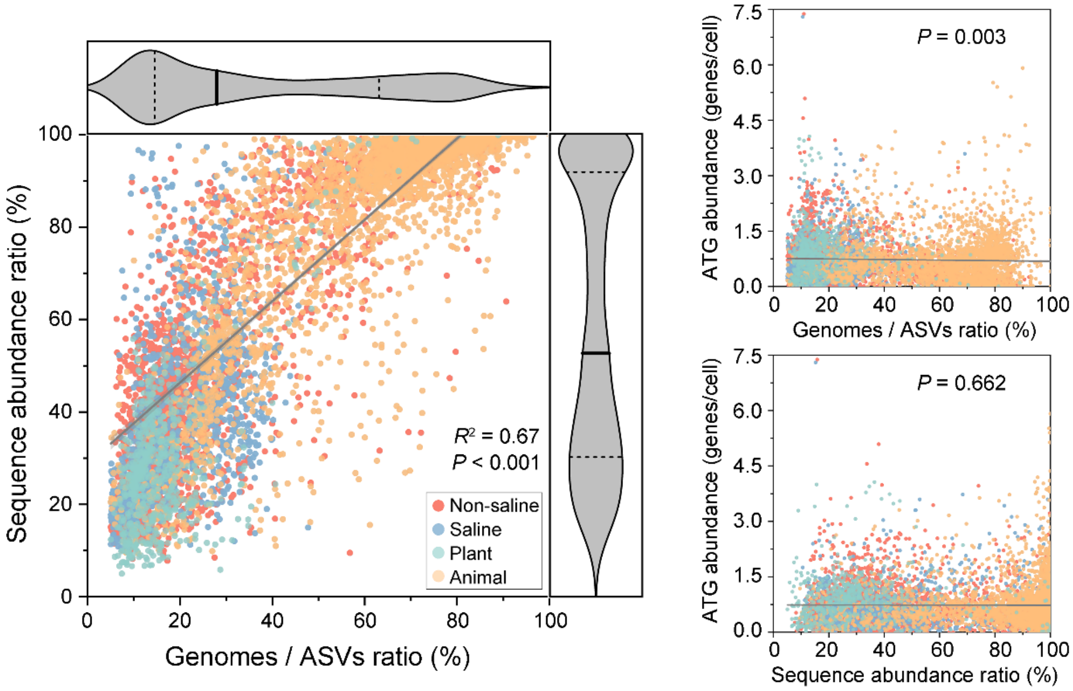

Fig. S1: Sequenced proportion of global microbial community genomes independent of ATG abundance. By mapping 262,011 ASV sequences and 217,614 RefSeq genomes at a threshold of 100% sequence identity, the median proportion of cells and taxa with known genome information in the studied EMP samples was 52.5% (30.1%-91.6%) and 27.9% (14.5%-62.9%), respectively. Each dot represents a community, with colors indicating different habitat types. The gray line indicates the best linear fit, and the shaded area represents the 95% confidence interval of the fitted curves. The middle line inside the violin plots shows the median, and the dashed line represents the 25th-75th percentiles.

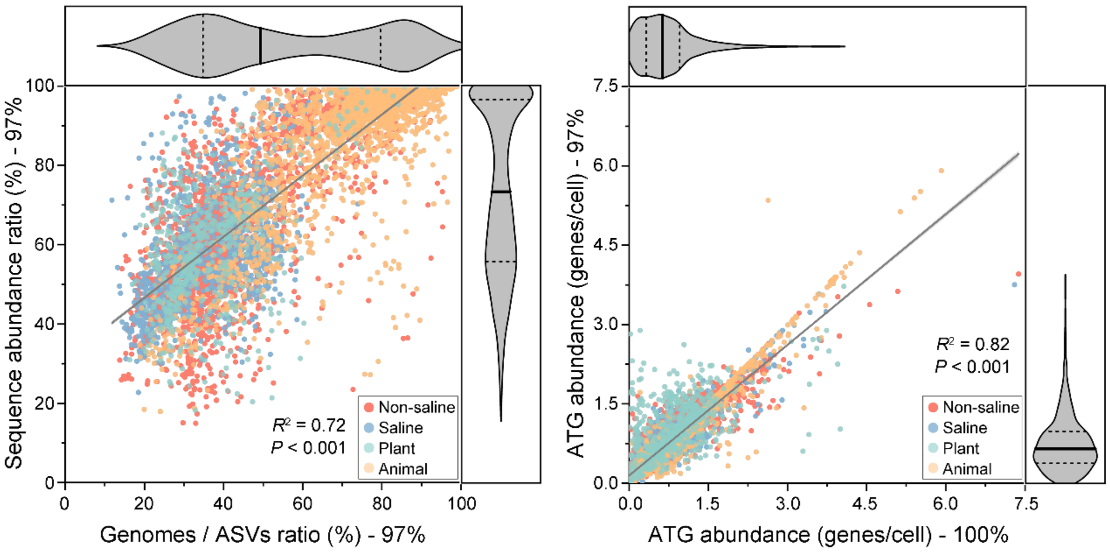

Fig. S2: Highly positive correlation of ATG abundances calculated at two mapping thresholds (97% and 100%). At a 97% sequence identity threshold, the median proportions of cells and taxa with known genomic information in the studied samples reached 73.2% (55.7%-96.3%) and 49.2% (34.7%-79.4%), respectively. Each dot represents a community, with colors indicating different habitat types. The gray line indicates the best linear fit, and the shaded area represents the 95% confidence interval of the fitted curves. The middle line inside the violin plots shows the median, and the dashed line represents the 25th-75th percentiles.

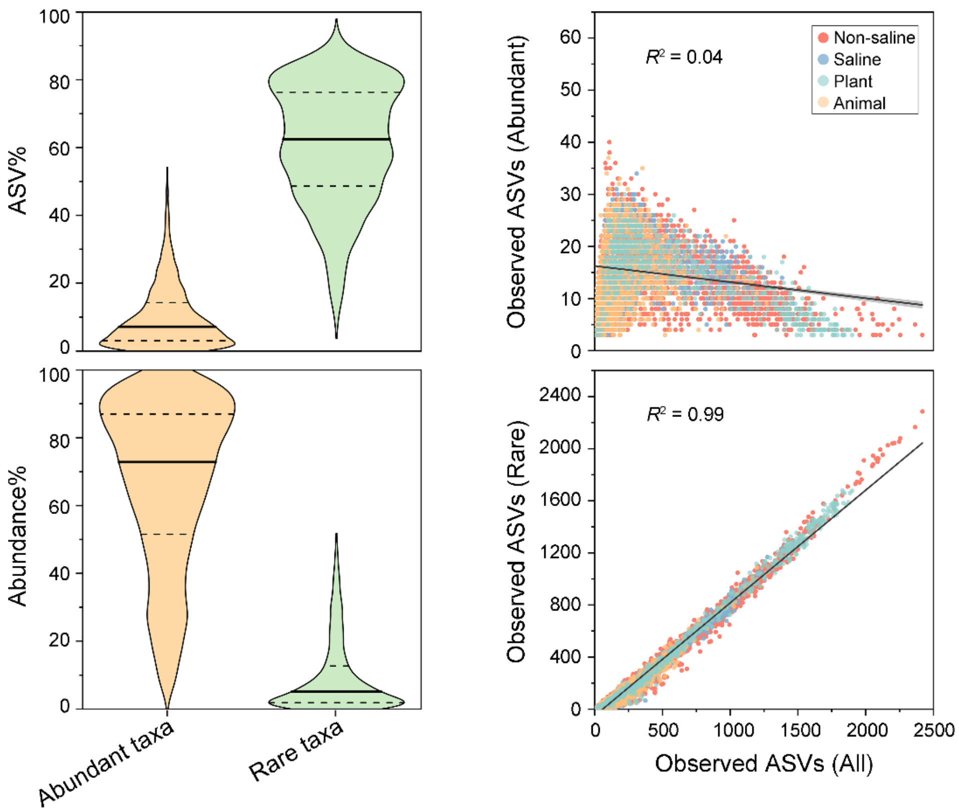

Fig. S3: Microbial communities consisting of few high-abundance abundant taxa and many low-abundance rare taxa. ASVs with a relative abundance ≥ 1% within a sample were defined as abundant taxa, whereas ASVs with a relative abundance < 0.1% were defined as rare taxa. The middle line inside the violin plots shows the median, and the dashed line represents the 25th-75th percentiles. Each dot represents a community, with colors indicating different habitat types. The gray line indicates the best linear fit, and the shaded area represents the 95% confidence interval of the fitted curves.

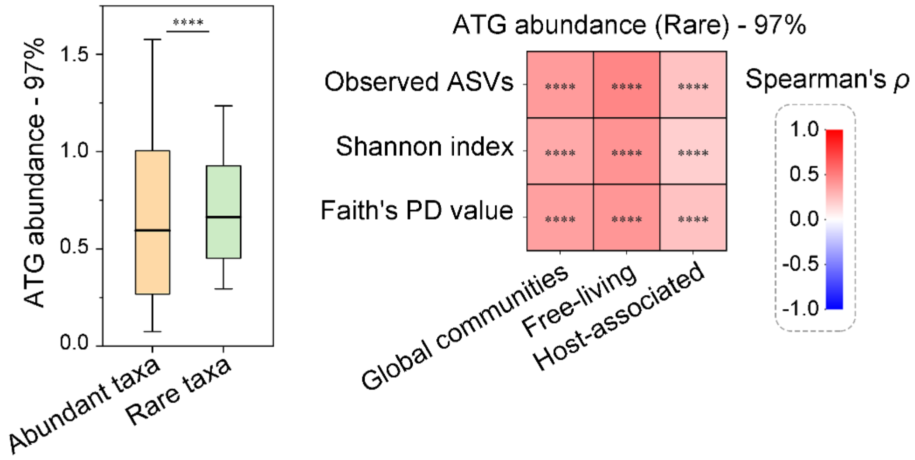

Fig. S4: At the 97% mapping threshold, the ATG abundance of rare taxa was significantly greater than that of abundant taxa, and the ATG abundance of rare taxa was significantly positively correlated with community biodiversity. Although the 97% threshold matched more ASVs than the 100% threshold, both thresholds yielded the same conclusions. For the box plots, the middle line indicates the median, the box represents the 25th-75th percentiles, and the error bar indicates the 10th-90th percentiles of observations. Comparisons between bins were analysed via the Wilcoxon signed-rank test. Correlations for the analyses were calculated separately based on global microbial communities, free-living communities, and host-associated communities. Biodiversity indices include observed ASVs, Shannon index, and Faith's PD value. The color scale indicates positive (red) or negative (blue) correlations, as determined by Spearman's ρ correlation coefficient. ****$P < 0.0001$.

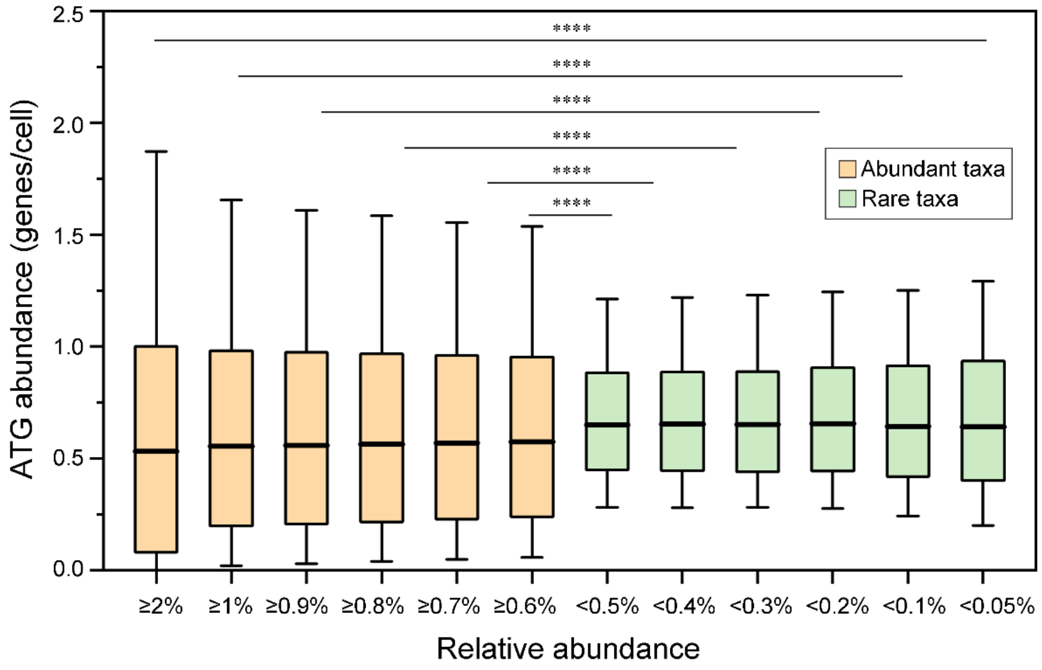

Fig. S5: ATG abundance of rare taxa was significantly greater than that of abundant taxa across different abundance thresholds. Six groups were calculated for abundant taxa (abundance ≥ 2%, ≥ 1%, ≥ 0.9%, ≥ 0.8%, ≥ 0.7%, or ≥ 0.6%) and rare taxa (abundance < 0.05%, < 0.1%, < 0.2%, < 0.3%, < 0.4%, or < 0.5%). For the box plots, the middle line indicates the median, the box represents the 25th-75th percentiles, and the error bar indicates the 10th-90th percentiles of observations. Comparisons between bins were analysed using the Wilcoxon signed-rank test. ****$P < 0.0001$.

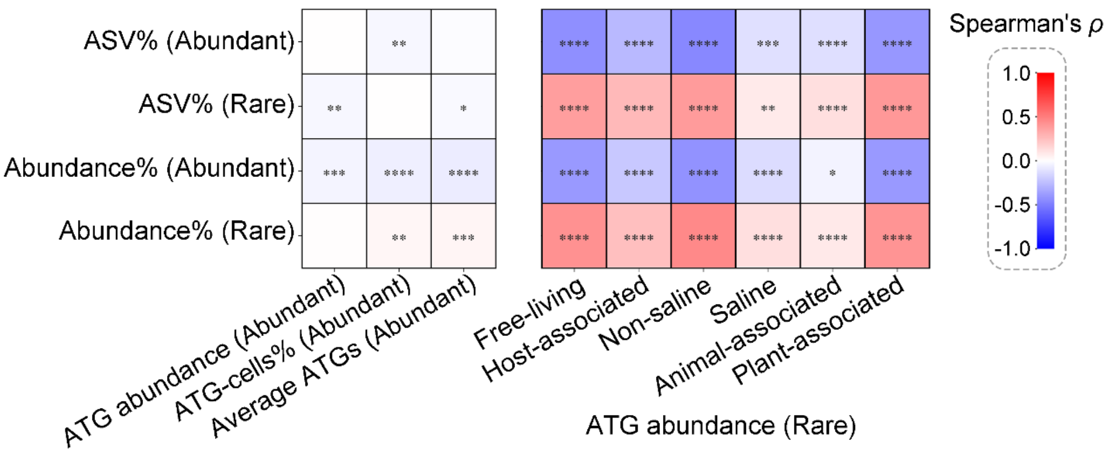

Fig. S6: The impact of rare taxa ATG abundance on community composition was strong, whereas that of abundant taxa was weak. Global microbial communities were classified as free-living and host-associated, with further subdivision into four types. The color scale indicates positive (red) or negative (blue) correlations, as determined by Spearman's ρ correlation coefficient. *$P < 0.05$; **$P < 0.01$; ***$P < 0.001$; ****$P < 0.0001$.

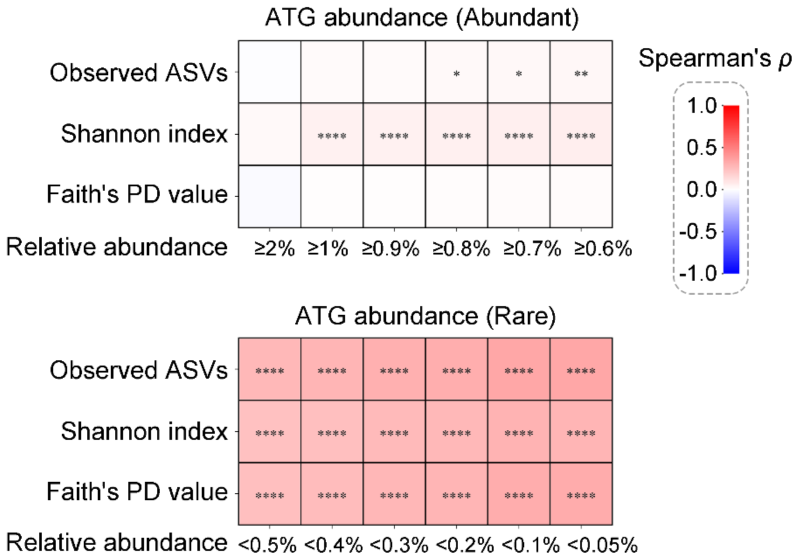

Fig. S7: Under different abundance thresholds, community biodiversity was significantly positively correlated with the ATG abundance of rare taxa but had no or weak correlation with the ATG abundance of abundant taxa. Six groups were calculated for abundant taxa (abundance ≥ 2%, ≥ 1%, ≥ 0.9%, ≥ 0.8%, ≥ 0.7%, or ≥ 0.6%) and rare taxa (abundance < 0.05%, < 0.1%, < 0.2%, < 0.3%, < 0.4%, or < 0.5%). Biodiversity indices are described as observed ASVs, Shannon index, and Faith's PD value. The color scale indicates positive (red) or negative (blue) correlations, as determined by Spearman's ρ correlation coefficient. *$P < 0.05$; **$P < 0.01$; ***$P < 0.001$; ****$P < 0.0001$.

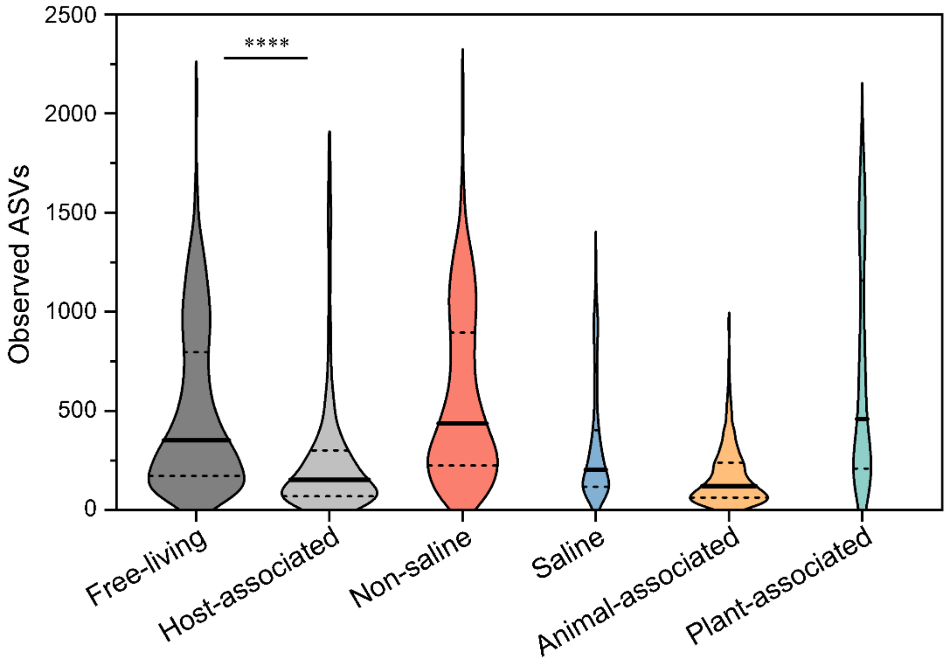

Fig. S8: Differences in the biodiversity of microbial communities among different habitat types. Biodiversity is described as the number of observed ASVs. The middle line inside the violin plots shows the median, and the dashed line represents the 25th-75th percentiles. Comparisons between bins were analysed using the Wilcoxon rank-sum test. ****$P < 0.0001$.

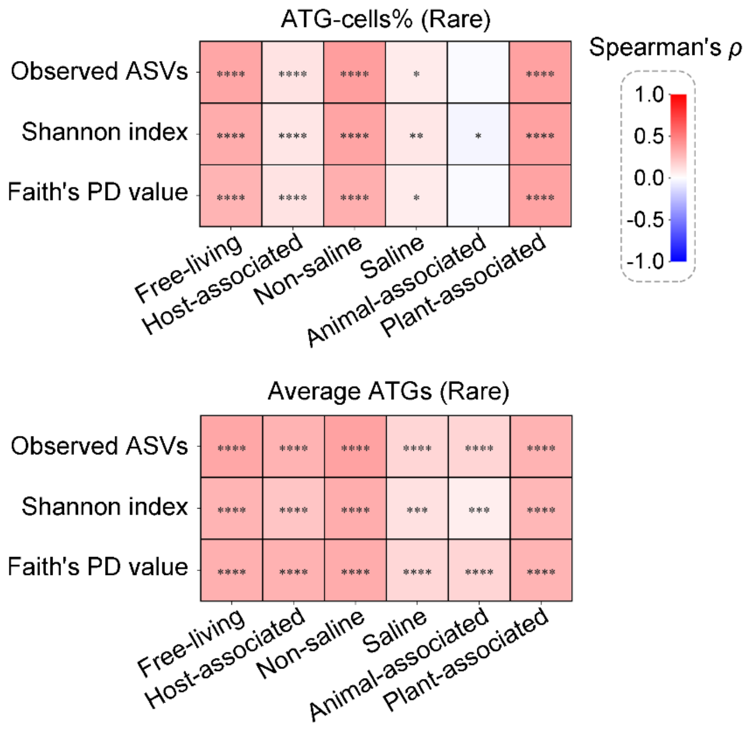

Fig. S9: Proportion of ATG-encoding cells in all cells and average number of ATGs per ATG-encoding cell in rare taxa were positively correlated with microbial community biodiversity. Global microbial communities were classified as free-living and host-associated, with further subdivision into four types. Biodiversity indices are described as observed ASVs, Shannon index and Faith's PD value. The color scale indicates positive (red) or negative (blue) correlations, as determined by Spearman's ρ correlation coefficient. **P* $<$ 0.05; ***P* $<$ 0.01; ****P* $<$ 0.001; *****P* $<$ 0.0001.

Supplementary Tables

Table S1. **Information on the identified ATGs in the microbial communities.**

Table S2. **Distribution data of ATGs in microbial communities.**

Table S3. ATG abundance of rare and abundant taxa defined by different thresholds.
